# Applications of cerebrospinal fluid circulating tumor cells and circulating tumor-derived DNA in diagnosis, prognosis, and personalized treatment of CNS metastases

**DOI:** 10.3389/fonc.2024.1409383

**Published:** 2024-09-30

**Authors:** Elena Pentsova

**Affiliations:** Department of Neurology, Memorial Sloan Kettering Cancer Center, New York, NY, United States

**Keywords:** brain metastasis, leptomeningeal metastasis, liquid biopsy, cerebrospinal fluid, biomarkers

## Abstract

A common feature of advanced solid tumors is their ability to metastasize and colonize distant organs, including the Central Nervous System (CNS), which encompasses brain and leptomeningeal metastases (LM). While cerebrospinal fluid cytopathological analysis remains a gold standard diagnostic tool, it only provides limited insights into the biology of tumor cells; thus, it is urgent to develop minimally invasive biomarkers that enable a comprehensive quantitative and molecular characterization of disseminated cells, therapy response assessment, and disease monitoring. Liquid biopsy methods have been swiftly developed for some readily accessible bodily fluids such as plasma and urine; circulating tumor cells (CTCs) and circulating tumor DNA (ctDNA) from these sources have been rapidly implemented into clinical trial design, disease monitoring, and treatment assignment across different tumor types. However, the filter imposed by the brain blood barrier (BBB) hampers the release of tumor-derived cells and molecules from CNS metastases. Crucially, cerebrospinal fluid (CSF) liquid biopsy methods offer a unique and unparallel source to develop liquid biopsy methodologies in patients with CNS-disseminated disease, including the characterization of CTCs and ctDNA arising specifically from brain and leptomeningeal metastasis. These technologies have enabled a deeper understanding of tumor cell and molecular dynamics, including the reconstruction of clonal evolution in the brain microenvironment through longitudinal sapling. Here, we discuss the current challenges and opportunities that CSF liquid biopsy methods face for the implementation of these approaches into clinical settings.

## Introduction

**Figure 1:**
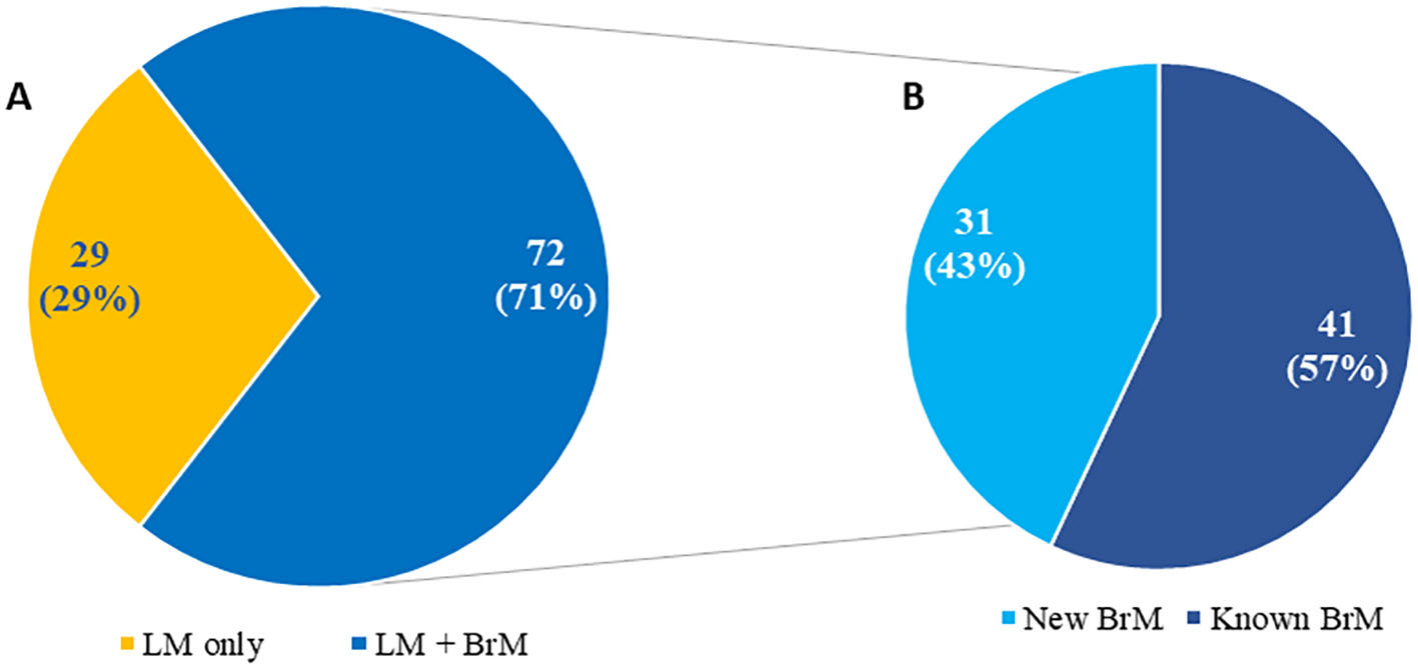
Distribution of patients (N = 101) with newly diagnosed leptomeningeal metastasis based on isolated leptomeningeal metastasis versus leptomeningeal metastasis and brain metastases **(A)** including time of brain metastases diagnosis **(B)** Adapted from Diaz’s paper “Quantitative assessment of circulating tumor cells in cerebrospinal fluid as a clinical tool to predict survival in leptomeningeal metastases” at Journal of Neuro-Oncology {Diaz, 2022 #256}.

Central Nervous System (CNS) metastases, encompassing brain metastases (BrM) and leptomeningeal metastases (LM), represent some of the most serious and common neurological complications of solid tumors. Among all tumor types, breast, lung cancers and melanoma, have the highest likelihood of developing CNS metastases (1–3). across CNS malignancies, the incidence of CNS metastases is 3-10 times higher than the incidence of primary brain tumors (4). More than 25% of patients with stage IV lung cancer may have BrM initially and up to 54% of these patients will subsequently develop BM following the primary tumor diagnosis (5–7). In breast cancer 5-20% of patients and in melanoma 7-16% of patients, developed BrM (8–11). Another devastating neurologic complication is LM whose incidence is challenging to determine at earlier stages based on current diagnostic criteria (12). LM can occur as a standalone disease without BrM, be present at the time of BrM diagnosis in up to 30% of patients or develop much later in patients with BrM (13) (**Figure 1**). The timing of LM diagnosis holds specific prognostic significance related to its detection (14).

Diagnostic methods for CNS metastases include neuroimaging, brain tissue diagnosis if surgical resection is feasible and cerebrospinal fluid (CSF) studies for LM diagnosis. However, resection of CNS metastases can be challenging to perform due to the eloquent location of these tumors and may not be indicated when multiple metastases are present. Interestingly, a recent study of resected BrM suggested that sequencing of primary tumors alone might miss molecular aberrations present in 53% of CNS metastases, and various drug resistance mutations could be identified in CSF in about 50% of patients with CNS relapse during various kinase inhibitor therapy. This indicates that a primary tumor biopsy alone might not be sufficient to achieve optimal molecular guidance to treat CNS metastases in advance tumors (15, 16). As systemic therapies improve, patients with cancer have longer survival prospects and higher chances of developing CNS metastases as tumors acquire treatment resistance and relapse/recur over time. Our understanding of the molecular landscape of CNS metastases remains limited at the time of BrM and LM diagnosis, throughout the disease course, and during treatment. Hence, there is a substantial clinical need to discover cerebrospinal fluid (CSF) biomarkers for the diagnosis, prognosis and assessment for treatment response in patients with CNS metastases.

### Leptomeningeal metastasis

LM is one of the underdiagnosed complications of cancer despite now being a treatable disease (12, 17–21). Establishing the diagnosis of LM based on standard cerebrospinal fluid (CSF) cytologic analysis or MRI findings is often difficult, particularly at the early stages of leptomeningeal dissemination when treatment interventions could be more effective in limiting spread and triggering additional symptoms. Brain and spine MRIs have the advantage of being non-invasive, but their findings may be nonspecific and equivocal for LM (22). CSF cytology examination is a “gold standard” minimally invasive diagnostic test that provides confirmation of LM but has low diagnostic sensitivity, often requiring multiple lumbar punctures to establish a diagnosis of LM (23–25). The interpretation of CSF cytology results in a dichotomous qualitative variable, positive or negative for malignant cells, and can be challenging if reported as suspicious or atypical. The current EANO/ESMO LM group consensus interprets those results as equivocal (26) and thus, negative results could be false negative as a result of low disease burden in the CNS compartment.

**Figure 2:**
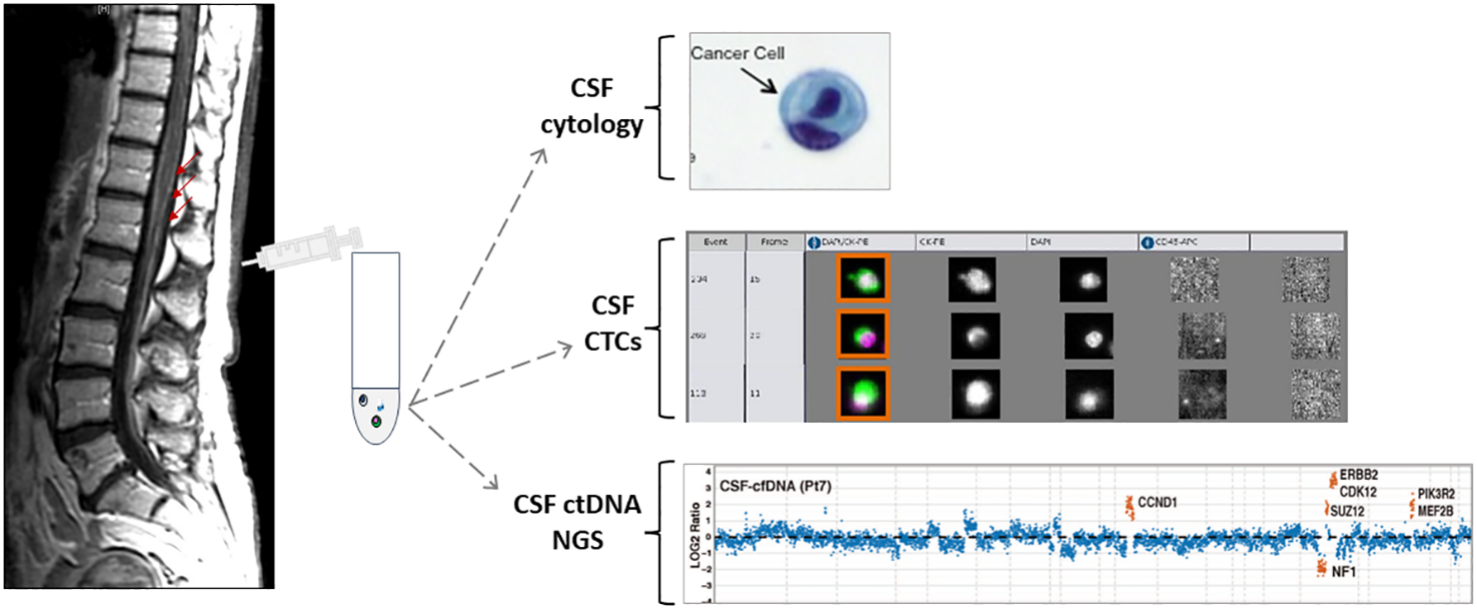
Schematic presentation of MRI of the spine with leptomeningeal metastasis and CSF collection followed by different CSF applications. Adapted from {Pentsova, 2016 #170} {Lin, 2017 #220}.

Recent development of new technologies and studies showing the feasibility to identifying tumor- derived cell-free circulating tumor DNA (ctDNA) in CSF and capturing and quantifying CSF circulating tumor cells (CTC) using rare cell capture technologies. This provides a unique opportunity to improve LM diagnosis and to characterize the molecular landscape of the CNS metastases, with the hope that “liquid biopsy” of CSF will be diagnostic, prognostic and therapeutic biomarkers (**Figure 2**).

### Quantitative assessment of disease burden in CSF: CSF CTC analysis by rare cell capture technologies

**Figure 3:**
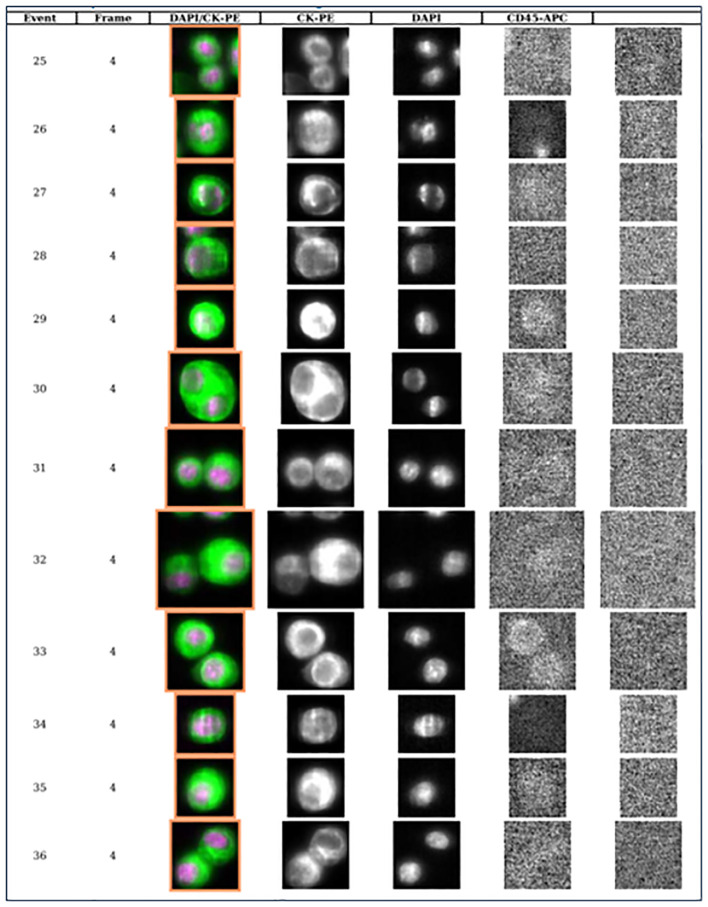
Library of CSF circulating tumor cells from patient with breast cancer and leptomeningeal metastases demonstrating 12 circulating tumor cells per 3 ml of CSF (each row represents one event or one CTC).

The validated CellSearch system (Menarini Silicon BioSystems), utilizing an immunomagnetic CTC selection method based on an rare cell capture technology (RCCT) and anti-epithelial cell adhesion molecule (EPCAM) antibody-conjugated ferroparticles, is an FDA-approved methodology for enumerating CTC from blood in patients with breast, prostate and colon cancers (27–31). The CellSearch system has been deployed to evaluate CSF-CTCs in patients with LM and has demonstrated potential as a diagnostic marker (**Figure 3**). A recent prospective study enrolled 95 patients with solid tumors (36 patients with breast cancer, 31 with lung cancer, 28 patients with other solid tumors) presenting with neurologic symptoms suspicious for LM or with MRI findings suspicious for LM, and referred them for clinical CSF examination (32). All patients underwent MRI of the brain and/or spine, and CSF cytopathology. Additionally, 3 ml of CSF from the same lumbar puncture were submitted for CSF-CTC analysis. Based on ROC analysis, presence of ≥ 3 CSF-CTC/per 3 ml was defined as the optimal cut-off for diagnosis of LM achieving a sensitivity of 93%, specificity 95%, positive predictive value 90%, negative predictive value 97%. Other studies reported similar findings of sensitivity above 80% and specificity above 95% (33–38). Additionally, several retrospective studies have demonstrated that LM patients with higher CSF-CTC counts at the time of diagnosis would have worse survival rate compared to those with lower counts, indicating a prognostic role for CSF-CTCs (39, 40).

### The role of CSF CTC analysis in response assessment

Advances in CSF biomarkers analysis allowed to further incorporate the CSF-CTC analysis as an exploratory endpoint in multiple IRB -approved clinical trials (19, 41, 42). For instance, in a phase I/II trial of intrathecal trastuzumab for LM in human epidermal growth factor receptor 2 positive (HER2+) breast carcinoma (42) aimed at characterizing changes in CSF CTCs over time as a potential biomarker of treatment response. Of the 15 patients, 9 had greater than 1 cycle of treatment and were evaluated: a decrease in CSF-CTC count with treatment, and a later increase in CTCs predating radiographic LM progression, were seen in several patients, which led to hypothesis that CSF-CTC analysis may serve as a platform to assess quantitative treatment response and should be further investigated.

The largest prospectively collected case series of 58 patients (41) treated with proton cranio-spinal irradiation (pCSI) reported statistical significance association (HR: 1.05 per 10 cells (95%CI: 1.01–1.10, *P* = .01) of pre-pCSI CSF-CTCs with CNS progression free survival (PFS) but not associated with overall survival. Moreover, in 31/58 patients with both a pre- and post-pCSI, no increase in CSF-CTCs was observed immediately post-pCSI. It is probably too early to conclude whether CSF-CTC results should influence the decision to discontinue treatment as the relationship between number of CSF-CTCs and radiographic disease CNS burden needs to be investigated further.

Currently the RCCT immunomagnetic platform for enumerating CTC in CSF could be available in a few but not all highly specialized cancer centers, complicating the widespread implementation of this approach, and the validation of its clinical utility across other institutions in the US and worldwide. Other limitations of the CellSearch platform are that it only detects cells from tumors of epithelial origin and have limited ability to provide comprehensive genomic characteristics of the cells. Thus far genomic sequencing of isolated CSF-CTCs has been achieved in patients with LM from breast cancer expressing human epidermal growth factor receptor 2 (HER2) and demonstrated similarities between CSF-CTCs and tumor cells from primary site in addition to several genetic alterations private to CSF-CTC (38, 42–44).

### Clinical significance of CSF ctDNA as a diagnostic, prognostic and treatment response biomarkers

Detection of ctDNA in plasma has showed promising potential as a source of “liquid biopsy” to reflect extracranial treatment response and outcomes in a variety of cancers (45–47). However recent studies have shown that plasma ctDNA may not accurately reflect intracranial disease burden in patients with BM (48, 49). Less than a decade ago several studies showed the feasibility of CSF cfDNA sequencing (16, 48–50). Several retrospective studies have demonstrated a positivity rate of CSF ctDNA consistently above 50% for patients with primary or metastatic brain tumors, including detection of tumor-associated mutations in the CSF of 63% of patients with brain metastases (16); in patients with glioma, CSF ctDNA can be found at a much higher positivity rate than plasma and correlates with prognosis (51, 52). CSF is relatively acellular and has direct anatomical contact with BM, making it easier to detect ctDNA in this fluid, potentially with a better correlation with prognosis than plasma ctDNA. Since than many groups reported results which suggested that CSF sequencing may offer a window into the identification of drug resistance mechanisms in patients whose primary tumor responded to genotype-directed targeted cancer therapy but then developed tumor progression in the CNS (**Table 1**). Additional work is needed to understand to what extent CSF cfDNA reflects the genetic alterations of the primary tumor and/or CNS metastases as a small study showed that some paired BM specimens had unique alterations in TP53 and KRAS, but there were notably very few unique mutations in the CSF specimens (65). Based on current data many questions need to be answered in a clinical setting: how to use CSF ctDNA to monitoring genetic evolution of the tumor in response to treatment, what the role of clonal and subclonal mutation in the CSF is, and whether CSF ctDNA presence or absence, or the percentage of variant allele frequency (VAL) in the sample are the marker of response and correlation with survival and might thus be clinically useful as a “surrogate” source of tumor-derived DNA.

**Table 1:** Studies analyzing CSF ctDNA in patients with solid tumors with and without brain and leptomeningeal metastases (Updated: July 2024).

Authors	N	Cancer Type	CSF ctDNA Sequencing Analysis
Pentsova E et al (16)	53	ST with +/-CNS mets, PBT	NGS
Cheok SK et al (53)	14	ST with CNS mets	Error-suppressed deep sequencing
Huang R et al (54)	35	ST with CNS mets	ddPCR
Ma C et al (55)	21	ST with CNS mets	NGS
Bale TA et al (56)	137pts 148samples	ST with CNS mets, PBT	NGS
Shen F et al (57)	77	ST with CNS mets: LM	NGS
Wang Y et al (58)	131	ST with CNS mets: LM	NGS
White MD et al (59)	22	ST with CNS mets: LM	WES
Wijetunga NA et al (60)	64pts 78samples	ST with CNS mets: LM	NGS
Choi W et al (61)	11	ST with LM	ddPCR and NGS
Pan et al (48)	8	ST with LM and CNS mets, PBT	Amplicon-based cancer gene panel sequencing, Digital PCR&targeted amplicon sequencing
Shah M et al (62)	38	ST with LM and CNS mets, PBT	NGS
Zhao Yue et al (63)	35	ST with LM, CNS lymphoma	NGS
De Mattos-Arruda L et al (49)	12	ST with LM, PBT	NGS
Fitzpatrik A et al (64)	24	ST: Breast/LM	NGS
Skakodub A et al (65)	13	ST: NSCLC LM +/-	NGS

Mets, Metastasis; LM, Leptomeningeal metastasis; ST, Solid tumors; PBT, Primary brain tumor; ddPCR, droplet digital polymerase chain reaction; NGS, Next-generation sequencing; WES, Whole-exome sequencing; CNS, Central Nervous System.

Molecular profiling of CSF in patients with brain tumors to identify potential micro RNAs (miR) associated to brain or leptomeningeal dissemination is of great interest, as it could provide a tool to predict and anticipate metastatic disease. Due to technical limitations in discerning tumor and immune cell miR profiles due to the rarity of CTCs, results are heterogeneous and require cautious interpretation. Despite heterogeneous findings, some studies report associations of specific miRs found in the CSF with the presence of brain tumors. An early study in patients with primary and metastatic brain tumors found, using a candidate approach, that miR10b, miR-21 and miR-200 were overexpressed in samples from patients with primary and metastatic brain tumors compared to controls{Teplyuk, 2012 #299}. Follow-up studies conducting miR profiling found differential expression of miR-30e, miR-140, let-7b, mR-10a and miR-21-3p in patients with brain tumors in a cohort of 175 patients that included 13 patients with metastasis; these miRs were validated in an external cohort of 105 patients {Kopkova, 2019 #298}. More recent approaches study miR signatures, which could be more informative as they aggregate global miR profile changes; in a cohort of 65 patients (including 11 patients with LM and 6 patients with BM), miR-335-5p and miR-34b-3p were linked to brain metastasis{Im, 2021 #297}. Although provocative, these studies are limited by the small number of patients, and most of them are not tested in prospective settings; besides these low numbers, these cohorts are heterogeneous as metastases from different types of primary tumors are often grouped to compensate the small cohort samples in granular analyses. Thus, future studies in this line should focus on larger, homogeneous cohorts and incorporate to the extent possible prospective testing of miR profiles.

Paralleling the advances in plasma and other non-CNS bodily fluids, considerable progress has been achieved after almost a decade of CSF-based liquid biopsy: from its conceptual inception and feasibility to its clinical validation, including the potential to influence clinical trial design, several roadblocks have been cleared out regarding the utility of CSF liquid biopsy for CNS disease. There are a few additional questions that must be answered to better understand the prognostic implications of detectable CTCs and ctDNA in CSF, the role of the molecular profile of CSF cfDNA from patients with CNS metastases and its correlation with the molecular profile of blood cfDNA/primary tumors or other sites of metastases to identify mutations or clonal populations that may predispose to CNS metastases. Most prominently, randomized prospective studies are sorely needed to provide sufficient evidence of feasibility in clinical pipelines, and a thorough benchmarking of molecular profiling methods (including cell detection and capture methods) has to be conducted to circumvent technical limitations of these approaches.

## Discussion

The advent of increasingly effective diagnostic procedure and treatments across cancer types has resulted in overall improved clinical outcomes for primary tumors, but CNS metastases are still a common feature for most advanced solid tumors, for which scanty treatment options are currently available. The treatments for CNS metastases often are assigned based on the molecular profiles of the primary tumor, and monitoring tumor responses remains a challenge beyond imaging, given that repeated biopsy is not always feasible or safe for these patients. This particularity of CNS metastasis severely limits the development of experimental approaches, for which response assessments are challenging. Besides, patients with CNS metastases are often excluded from trials because of severe neurological deficits.

In contrast to most other solid tumors, traditional liquid biopsy approaches using plasma have been largely ineffective for CNS-disseminated disease, due to the limited release of cellular and molecular tumor components across the brain-blood barrier towards the main circulatory system. Thus, CSF-based liquid biopsies have profoundly revolutionized the ways in which we can access to these types of clinically relevant information in a minimally invasive manner.

The development of CSF biomarkers may allow us to diagnose CNS metastases earlier and find why CNS disease does not respond to treatment at the time of recurrence, thus increasing the chance patients can participate in clinical trials.

As these methods continue to develop with increasing resolution, sensitivity, and specificity, data integration with orthogonal methods (such as electronic health records, advanced imaging, patient-reported outcomes, and computational methods) will increase the robustness of CSF liquid biopsy as a useful biomarker that provide unique clinical and molecular information.
